# Mobile Phone Addiction, Phubbing, and Depression Among Men and Women: A Moderated Mediation Analysis

**DOI:** 10.1007/s11126-020-09723-8

**Published:** 2020-03-07

**Authors:** Ana Ivanova, Oleg Gorbaniuk, Agata Błachnio, Aneta Przepiórka, Natalia Mraka, Viktoria Polishchuk, Julia Gorbaniuk

**Affiliations:** 1 John Paul II Catholic University of Lublin, Lublin, Poland; 2 Uniwersytet Zielonogórski, Góra, Poland; 3 L’viv State University of Internal Affairs, Lviv, Ukraine; 4 National University Ostroh Academy, Ostroh, Ukraine

**Keywords:** Phubbing, Mobile phone addiction, Depression, Loneliness, Moderated mediation

## Abstract

For several years, the number of studies on the links between excessive mobile phone use and mental health has been increasing. The aim of the study was to establish if there is a relationship between mobile phone addiction and depression in university students and if phubbing is a mediator of this relationship. The authors also tested if this mediation effect was moderated by loneliness and if the model of relationships between these variables was the same in women and in men. The participants were 402 university and college students from Ukraine, aged 17 to 31; 74% of them were women. The authors used the Adapted Mobile Phone Use Habits, the Phubbing Scale, the Center for Epidemiologic Studies Depression Scale, and the Loneliness Scale. The results of the study have shown that higher mobile phone addiction and higher phubbing is associated with a higher level of depressive moods, with phubbing functioning as a mediator of the relationship between mobile phone addiction and depression. A moderator of this mediation is loneliness, the moderation effect being asymmetrically dependent on gender: in men, high loneliness increases the mediating role of phubbing, which more markedly translates into depression, while in women the analyzed mediation effect becomes weaker with an increase in the sense of loneliness (phubbing correlates less strongly with depression).

## Introduction

The number of studies reporting detrimental effects of smartphones on mental and physical health is constantly growing [4, 18, 31, 37]. The negative consequences for health in the group of phone and smartphone^1^ addicts are similar to those of Internet and gaming addiction [6, 40]. The aim of the study was to establish if there is a relationship between mobile phone addiction and depression in university students and if phubbing is a mediator of this relationship. We also tested if this effect was moderated by loneliness and if the model of relations between these variables was the same for women and for men.

### Mobile Phone Addiction

Although in the literature there is a debate on whether excessive mobile phone use can be understood as a behavioral addiction [11, 27], and although the same phenomenon is referred to by different terms, such as problematic cell-phone/mobile phone use [10, 33, 62], cell-phone overuse [50], mobile phone dependence [19], or mobile phone/smartphone addiction [21, 35, 58, 60], problematic mobile phone use is often conceptualized as a behavioral addiction, characterized by the basic symptoms of addictive behaviors [19, 41, 42, 64]. Regardless of the ongoing debate, smartphone addiction is a spreading phenomenon especially among young people due to their increased social communication frequency, their greater opportunities for making social relationships, and their better skills in using modern technological devices [45]. Although many studies show that women more often have a tendency to use the mobile phone in a problematic way that men [17, 33, 55, 62], there is also evidence that in the group of adolescents men may spend more time using smartphones compared to women [28] or that women and men are at the same risk of problematic smartphone use [7, 22]. Moreover, the results of the studies conducted to date show that smartphone addiction is positively linked with health problems [33, 56], psychological problems [59], and psychiatric disorders, particularly depression [1, 26]. Loneliness is a predictor of smartphone addiction as well [9, 67].

### Phubbing

The term “phubbing” is a merger of two words: “phone” and “snubbing.” It was coined as part of the Macquarie Dictionary campaign by a group of lexicographers, poets, and authors [48] in order to name the phenomenon of snubbing the interlocutor in the context of social contact by devoting one’s attention to the mobile phone instead of focusing on the person one is talking to.^2^ Phubbing manifests itself in focusing on the phone while talking to a person whom the contents in the phone do not concern [20, 35, 47]. Previous studies have shown that phubbing is positively correlated with mobile phone addiction [20, 35], which in turn is used as a tool helping in situations of loneliness, anxiety, and worry, and with deprivation in situations of being far from one’s phone [35]. Phubbing, however, not only does not help but actually aggravates phubbers’ problems: smartphones increase the sense of exclusion from the social environment during smartphone use [36]. Phubbed individuals experience social exclusion too, which in turn increases their need to be in the center of attention in their important reference groups and induces them to use social media in order to reduce the sense of exclusion [23]. Research shows that phubbing is positively correlated with loneliness [12], understood as protracted and negatively valenced feeling of social exclusion [49]. Moreover, previous studies have shown that gender can be a moderator of the relationships between phubbing and mobile phone, SMS, social media, and Internet addictions; in the female group, phubbing was related to mobile, SMS, and social media addictions, whereas in the male group it was associated with Internet and gaming addictions [35], with women usually scoring higher than men on phubbing [3, 12, 20]. This means that in the female group smartphones are more often used to facilitate social interactions, whereas in the male group mobile phones perform instrumental functions [4, 55]. As is the case with smartphone addiction, phubbing is related to mental health: indirect and direct positive associations have been found between phubbing and depression in various age groups [22, 54, 63].

### Depression and Loneliness

Depressive disorders manifest themselves in sadness, a sense of emptiness, or irritability; these symptoms are accompanied by somatic and cognitive changes significantly affecting the individual’s functioning [43]. Also stress plays an important role in the emergence of depression [8]. Studies show that people who more often experience depressive symptoms more often suffer from social media addiction; for instance, it has been found that depression is positively related to Facebook addiction [13], mobile phone addiction [1, 26], and phubbing [22, 54, 63]. Moreover, studies show that there is a positive relationship between depression and the sense of loneliness [5, 15, 53]. Ayas and Horzum [2] found that loneliness as well as depression and self-esteem were related to Internet addiction. It has also been established that loneliness is a positive predictor of Facebook addiction [14] and mobile phone addiction [62].

### Aim of the Study

**Fig. 1:**
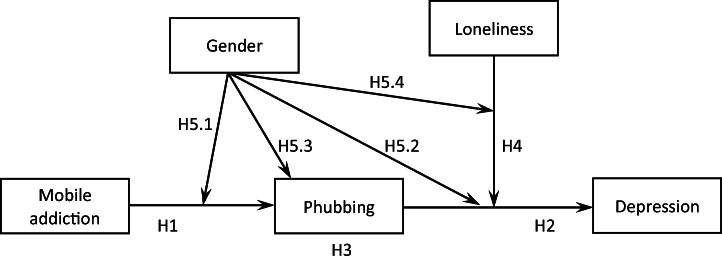
The conceptual model of the relationship between mobile phone addiction, phubbing, and depression

Phubbing is a relatively new phenomenon, which is why, to our knowledge, there are no studies in the literature so far investigating more complex relationships between mobile phone addiction, phubbing, depression, loneliness, and gender. However, based on the studies conducted to date, such as those in the field of new media psychology, it is possible to predict that relationships between these variables do exist. The aim of the present study was to determine the relationships between mobile phone addiction and depression, with phubbing as the mediator of this relationship. We also tested if this mediation effect was moderated by loneliness and if the model of relationships between these variables was the same in women and in men. To our knowledge, in previous studies little space was devoted to the role of gender as a moderator in the relations of phubbing to its determinants and antecedents, and it is these relations that constituted the main object of interest in our research. Figure 1 schematically presents the expected relationships. We tested the postulated model among mobile phone users in Ukraine, where the number of mobiles per 100 inhabitants is one of the highest in Central and Eastern Europe and stands at 135 [46]. According to statistics, 52% of people in Ukraine use the Internet, which means that this country ranks 110th among 208 countries in the world.^3^ We hypothesized that mobile phone addiction would be positively correlated with phubbing and depression (H1) and that phubbing would also be positively correlated with depression (H2). We predicted that the relationship between mobile phone addiction and depression would be mediated by phubbing (H3) and that the strength of this mediation would be moderated by loneliness (H4). Finally, we expected that the relationships between mobile phone addiction, phubbing, depression, and loneliness would be different in women and in men (H5). Another aim of the study was to check if there were differences between men and women in the levels of phubbing and mobile phone addiction in the population of Internet users in Ukraine.

## Method

### Participants and Procedure

The participants in the study were 402 university and college students from Ukraine, aged 17 to 31(*Me* = 20 years); 74.1% of them were women. As many as 91.1% of the students were single, 7.9% were married, and 1.0% were divorced. The mean reported Internet use time was *M* = 5.26 h per day on weekdays (*Me* = 4.5 h, *SD* = 3.55 h) and *M* = 5.77 h per day on weekends (*Me* = 5.0 h, *SD* = 3.66 h). The tested sample makes it possible to identify the correlation of *r* > .15 between the variables as well as differences of Cohen’s *d* > 0.30 between the means at the significance level of α = .05 with a power of 1 – β = 0.90. The research was conducted as an Internet survey, from April to June 2017. The link to the website with the questionnaires was sent out to potential respondents. The respondents answered the questions by clicking the appropriate response option. In the case of no answers, the server reminded them to go back and give the missing ones. The participants received no remuneration and were informed that their anonymity would be ensured.

### Measures

The measures used were translated into Ukrainian by two translators and then back-translated; the discrepancies in translation were discussed, and we chose the version that was the best measure of the operationalized variable.

#### Measurement of Mobile Phone Addiction

To measure mobile phone addiction, we administered the *Adapted Mobile Phone Use Habits* (AMPUH; [61]). It consists of 10 items (example item: *Do you lie to others about how much you use your cell phone?*), which the participants rate on a 5-point scale from 1 – *never* to 5 – *always*. The internal consistency of the original version of the AMPUH is α = .75. The internal consistency of the version adapted into Ukrainian was α = .65.

#### Measurement of Phubbing

To measure phubbing, we used the Phubbing Scale [35]. This instrument consists of 10 items (example item: *I’m busy with my mobile phone when I’m with friends*), which the subjects respond to on a 5-points scale from 1 – *never* to 5 – *always*. It comprises two dimensions: Communication Disturbance and Phone Obsession. A high score on the Communication Disturbance subscale means that, being preoccupied by his or her own mobile in other people’s presence, the person interrupts the process of face-to-face communication. A high score on the Phone Obsession subscale indicates that the respondent constantly needs to have a smartphone at hand when there is not enough face-to-face communication. The internal consistency of the former subscale in the original version of the measure is α = .87, and the internal consistency of the latter subscale is α = .85. The internal consistency of these subscales adapted into Ukrainian is α = .77 for Communication Disturbance and α = .72 for Phone Obsession.

#### Measurement of Depression

To measure depression, we used the short form of the Center for Epidemiologic Studies Depression Scale (S-CES-D) [29, 51], which consists of 10 items measuring the level of depression (e.g., *I had trouble keeping my mind on what I was doing. I had a crying spell*). The participants are asked how often they have felt in the ways described during the past week. They indicate their responses on a 4-point scale: *rarely or none of the time (less than 1 day)*, *some or a little of the time (1–2 days)*, *occasionally or a moderate amount of time (3–4 days)*, *most or all of the time (5–7 days)*. The internal consistency of the Ukrainian version of the scale was α = .80.

#### Measurement of Loneliness

To measure the level of loneliness, we used the De Jong Gierveld Loneliness Scale [24, 25], which consists of 11 items (e.g., *I miss having a really close friend*) rated on a 6-point scale. Depending on the study, the internal consistency of the original scale ranges between α = .80 and α = .90. The homogeneity of the measure also varies across studies, with Loevinger’s *H* ranging between .30 and .50 [25]. The internal consistency of the Ukrainian adaptation was α = .85.

### Statistical Analyses

The relationships between pairs of variables were assessed by means of Pearson’s *r* coefficient. To test the differences between the groups in the measurements carried out in the study, we performed a *t*-test. Moderation tests for the relationships between the variables and mediation tests were performed by means of Hayes’s [32] algorithm with bootstrapping.

## Results

Table 1 shows descriptive statistics and correlations between the variables: mobile addiction, phubbing (communication disturbance and mobile obsession), depression, and loneliness for the whole sample and for both sexes. We also checked if there were differences between men and women in terms of the variables measured in the present study. The results of the *t*-test showed that women and men did not differ significantly in terms of mobile phone addiction (*t*(400) = 0.32, *p* = .753), communication disturbance (*t*(400) = 1.41, *p* = .158), depression (*t*(400) = 0.81, *p* = .420), and loneliness (*t*(400) = 1.39, *p* = .166). We observed higher phone obsession in women (2.80 (*SD* = 0.86) vs. 2.47 (*SD* = 0.76), *t*(400) = 3.44, *p* < .001, *d* = 0.34).

**Table 1:** Pearson’s r correlations between the study variables for men, women, and the whole sample

			Descriptive stats		Pearson’s r correlation coefficients			
		Variables	*M*	*SD*	1	2	3	4
Whole sample	1	Mobile Addiction	2.00	0.53	–			
	2	Communication disturbance	1.90	0.60	0.49**	–		
	3	Phone obsession	2.72	0.85	0.47**	0.61**	–	
	4	Depression	2.11	0.58	0.19**	0.21**	0.18**	–
	5	Loneliness	3.03	0.77	0.19**	0.12*	0.02	0.49**
Men	1	Mobile Addiction	2.01	0.54	–			
	2	Communication disturbance	1.93	0.61	0.51**	–		
	3	Phone obsession	2.80	0.86	0.50**	0.61**	–	
	4	Depression	2.12	0.60	0.19**	0.21**	0.17**	–
	5	Loneliness	3.00	0.76	0.19**	0.12*	0.04	0.46**
Women	1	Mobile Addiction	1.99	0.50	–			
	2	Communication disturbance	1.83	0.59	0.44**	–		
	3	Phone obsession	2.47	0.76	0.35**	0.59**	–	
	4	Depression	2.07	0.53	0.19	0.18	0.21*	–
	5	Loneliness	3.13	0.79	0.19	0.12	0.01	0.59**

* *p* < 0.05, ** *p* < 0.01

We found that mobile phone addiction and communication disturbance were positively related to both depression and loneliness, while mobile obsession was positively related only to depression. The results of analyses also revealed that mobile phone addiction correlated with communication disturbance (*r* = .49, *p* < .001) and phone obsession (*r* = .47, *p* < .001): the higher the addiction, the higher the phubbing—which confirms hypothesis H1. Moderation effect testing revealed that gender did not moderate the relationship between these variables (communication disturbance: *F*(1, 398) = 0.19, *p* = .664; phone obsession: *F*(1, 398) = 2.62, *p* = .106), which means hypothesis H5.1 was not confirmed. In the whole sample we found a statistically significant relationship between phubbing and depression: communication disturbance caused by phone use (*r* = .21, *p* < .01) and phone obsession (*r* = .18, *p* < .01) translate into a higher level of depression, which confirms hypothesis H2. Also in this case gender was not a moderator of the relationship between these variables (communication disturbance: *F*(1, 398) = 0.13, *p* = .721; phone obsession: *F*(1, 398) = 0.13, *p* = .716), which means hypothesis H5.2 was not confirmed.

The next step was mediation and moderation analysis, in which we tested the mediation effect using the bootstrapping method. The analysis revealed that the relationship between mobile phone addiction and depression was mediated by communication disturbance (*b* = 0.065, *boot SE* = 0.034, 90% CI [0.013, 0.125]), whereas phone obsession was not a statistically significant mediator (*b* = 0.029, *boot SE* = 0.031, 90% CI [−0.021, 0.082]). This means that hypothesis H3, postulating the mediation of the relationship between mobile phone addiction and depression by phubbing was confirmed for communication disturbance. We also found that gender moderated the mediation effect neither in the case of communication disturbance (*index* = −0.057, *boot SE* = 0.076, 90% CI [−0.177, 0.068]) nor in the case of phone obsession (*index* = 0.035, *boot SE* = 0.066, 90% CI [−0.069, 0.148]). This means that hypothesis H5.3, postulating that gender is a moderator of the mediation of the relationship between mobile phone addiction and depression by phubbing, was not confirmed.

**Fig. 2:**
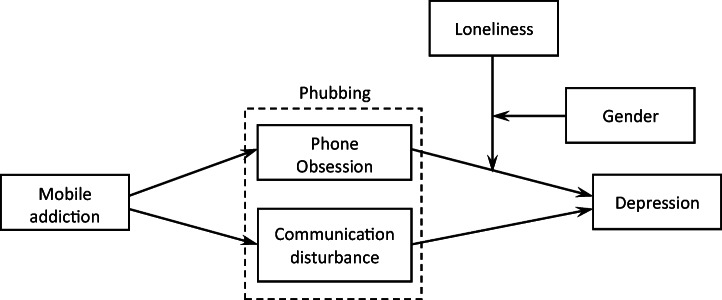
The final model of the relationship between mobile phone addiction and depression, with the mediating role of phubbing and the moderating role of loneliness and gender

Next, we tested if loneliness was a moderator of the mediation of the relationship between mobile phone addiction and depression (see Fig. 1). Test results showed that the moderated mediation was statistically significant neither in the case of communication disturbance (*index* = −0.048, *boot SE* = 0.038, 90% CI [−0.111, 0.014]) nor in the case of phone obsession (*index* = −0.001, *boot SE* = 0.037, 90% CI [−0.063, 0.059]). Finally, we tested if gender moderated the moderation of the mediation presented in Fig. 1 (hypothesis H5.4). Test results showed that gender was a statistically significant moderator of the moderation of mediation in the case of phone obsession (*index* = 0.147, *boot SE* = 0.083, 90% CI [0.013, 0.283]). This means that, in men, an increase in loneliness is accompanied by an increase in the mediating role of phone obsession in the intensification of depressive symptoms: the effect increases from *b* = 0.018 (*boot SE* = 0.058) at low loneliness to *b* = 0.143 (*boot SE* = 0.074) at high loneliness. In women, the direction of the moderation of mediation by loneliness is the opposite: at low loneliness, phone obsession mediates the relationship between mobile phone addiction and depression more strongly (*b* = 0.083, *boot SE* = 0.053), whereas at high loneliness the mediation effect disappears (*b* = 0.020, *boot SE* = 0.051). In the case of communication disturbance, the tested moderation was not statistically significant (*index* = −0.044, *boot SE* = 0.084, 90% CI [−0.181, 0.093]). This means that hypothesis H5.4. was confirmed in the case of phone obsession as a mediator. Figure 2 sums up the relationships found between the variables.

## Discussion

The main aim of the study was to establish if there is a relationship between mobile phone addiction and depression in university and college students and if phubbing is a mediator of this relationship. To our knowledge, this was the first study to investigate such relations between the variables. We found that the higher were the levels of mobile phone addiction and phubbing, the higher was the level of depressive moods; we also established that phubbing functioned as a mediator of the relationship between mobile phone addiction and depression. A moderator of this mediation is loneliness, the moderation effect being asymmetrically dependent on gender: in men, high loneliness increases the mediating role of phubbing, which more markedly translates into depression, while in women the analyzed mediation effect becomes weaker with an increase in the sense of loneliness (phubbing correlates less strongly with depression).

Mobile phone addiction is positively correlated with phubbing and depression (Hypothesis 1); phubbing is positively correlated with depression as well (Hypothesis 2). The obtained results are consistent with previous research reports: positive relationships have been observed between mobile phone addiction and depressive symptoms [1, 26], between phubbing and depression [22, 54, 63], and between mobile phone addiction and phubbing [20, 35]. The possible causes of the emergence of depression in a situation of Internet addiction were identified by Kraut et al. [38]: Internet use negatively affects well-being, which suggests that Internet addiction may lead to depression. Similar patterns have been detected in the case of smartphone use, with interpersonal relations additionally taken into account: phubbing indirectly increases the symptoms of depression by decreasing relationship satisfaction and life satisfaction [54]; moreover, partner’s phubbing indirectly contributes to depression by decreasing relationship satisfaction [63].

Mobile phone addiction increases the symptoms of depression through communication disturbance, but phone obsession is not a statistically significant mediator of this relationship (Hypothesis 3). The sense of loneliness moderates the strength of this mediation neither in the case of communication disturbance nor in the case of phone obsession (Hypothesis 4), which seems to be a surprising result in the context of the literature on the subject. The possible cause of the established patterns may be the relationship between phubbing, social exclusion, and depression. A high score on the Communication Disturbance subscale means that, being preoccupied by his or her mobile in other people’s presence, the person interrupts the process of face-to-face communication. The results of the studies conducted to date show that ignoring the interlocutor(s) by using a smartphone in their presence may increase the sense of exclusion from the social environment [36]. Social exclusion may in turn increase the symptoms of depression [39].

Since there are not many reports on the moderating role of gender differences in the literature, in the present study we tested if gender played the role of a moderator for the relationships between the variables. The testing of the role of gender as a moderator revealed that it did not moderate the relationship between mobile phone addiction and phubbing (Hypothesis 5.1). In several previous studies researchers tested the moderating role of gender in the relationship between mobile phone addiction and phubbing and obtained contradictory results. Karadağ et al. [35] found that gender moderated the relationship between mobile phone addiction and phubbing (the relationship was stronger for women than for men), while in the study by Chotpitayasunondh and Douglas [20] gender was not a statistically significant moderator of the same relationship. In our study, we also found that gender did not moderate the relationship between phubbing and depression (Hypothesis 5.2). In previous studies gender was not tested as a moderator of the relationship between phubbing and depression, but there have been attempts to detect associations between similar variables. For example, Sánchez-Martínez and Otero [59] established that, of many other variables, depression and gender (female) were predictors of problematic smartphone use [59].

When testing more complex relationships between the variables in the present study, we have established that gender does not moderate the mediating effect of phubbing in the relationship between mobile phone addiction and depression (Hypothesis 5.3). We have also established that in the case of communication disturbance the mediating role of phubbing is independent of the person’s gender and loneliness. In the case of phone obsession, by contrast, the mediating role of phubbing depends on the interaction of loneliness and gender (Hypothesis 5.4). In the case of lonely men, mobile phone addiction increases the symptoms of depression through phone obsession. In women, the mediating role of obsession is weaker and has the opposite direction: as loneliness increases, phone obsession ceases to mediate the relationship between mobile phone addiction and depression. These patterns can be explained in the light of the purposes for which women and men use smartphones. For men the smartphone is a device serving pragmatic functions, while for women it serves the purpose of contacting other people [4, 34, 55]. For this reason, mobile obsession – preoccupation with the phone when there is not enough face-to-face contact – in the group of women who feel lonely probably means communicating with others by means of the smartphone, which leads to a decrease in the sense of social exclusion and thus contributes to the weakening of depressive symptoms (cf. [39]). In the group of lonely men, by contrast, mobile obsession may more often manifest itself in the form of excessive Internet use or gaming, which in turn may increase the symptoms of depression [16, 38].

Research results have shown that women and men do not differ in the level of smartphone addiction. In the literature on the subject, the predominant results are those according to which women have a stronger tendency to use mobile phones in problematic ways compared to men [33, 62]. Our study is consistent with the results reported by Bianchi and Phillips [7] and by Davey et al. [22], who also found no differences between representatives of different genders in terms of problematic mobile phone use. Other studies, which revealed no differences between women and men, focused on selected aspects of mobile phone addiction: social networking on smartphones [57] or text-messaging dependency [44]. The cause of the absence of differences in smartphone addiction between women and men may be the fact that the question of the relationship between gender and addiction to technology remain open: although it is men rather than women who use the computer and the Internet in problematic ways more often [30, 65], the profile of a person experiencing problems associated with the use of technology changes with the passage of time [66]; women’s attitudes to technology also change, evolving towards more positive ones [52]. According to Bianchi and Phillips [7], the original gender differences are a product of socialization and access to technology; it is possible that with time these differences will begin to disappear.

The measurement of the level of phubbing also revealed no differences between women and men in scores on the Communication Disturbance subscale, and in the group of women we observed higher phone obsession. In previous studies in which the same measure was used, the results varied: Błachnio and Przepiórka [12] observed higher scores on both phubbing subscales in the case of women, whereas Davey et al. [22] found no differences whatsoever between women and men. A high score on the Phone Obsession subscale indicates that the respondent constantly needs to have a smartphone at hand when there is not enough face-to-face communication [35]. It is possible that the differences between women and men in the level of phone obsession stem from the fact that women perceive technological devices – such as smartphones or the Internet – mainly as communication tools, as opposed to men, for whom technological devices perform pragmatic functions (e.g., looking for information, entertainment) [4, 34, 55]. Therefore, when there is not enough face-to-face communication, women use smartphones for mediated communication more often than men do.

### Limitations and Future Studies

The study presented in this paper has several limitations. Firstly, the sample was narrowed down to university and college students. It is commonly known that smartphone users belong to various age groups, which means the problems of mobile phone addiction or phubbing may concern not only students—as has been demonstrated in the studies conducted to date [54, 63]. Therefore, our suggestion for the future is to test a sample representing the full age range in order to check how age moderates the established patterns. Secondly, the study followed a cross-sectional design, which means the obtained results cannot be interpreted as showing cause-and-effect relationships. Given the limitations of the present study, our suggestion for the future is to design and conduct a longitudinal study that will make it possible to look more closely into the relationships between smartphone addiction, phubbing, and depression. Thirdly, the presented study was conducted in Ukraine, and research should be extended to other countries in order to check if the relationships we have found are universal or specific to only some of the countries.

## Conclusion

To sum up, the main aim of this study was to determine the links between mobile phone addiction and depression in university and college students and to establish if phubbing is a mediator of this relationship. We also tested if this mediation effect was moderated by loneliness and if the model of relationships between these variables was the same in women and in men. We have found that mobile phone addiction and phubbing are positively associated with depression, with phubbing functioning as a mediator of the relationship between mobile phone addiction and depression. A moderator of this mediation is loneliness; interestingly, the moderation effect is asymmetrically dependent on gender. The present study is a step on the way towards a better understanding of the phenomena of phubbingu and mobile phone addiction. Above all, its results point to the role of gender in the emergence of both phenomena and may inspire further research.

## Footnotes

1 In this project, we use the terms “smartphone” and “mobile” interchangeably. Whenever we say “mobile” we think about a phone with access to the Internet.

2 http://stopphubbing.com

3 https://en.wikipedia.org/wiki/List_of_countries_by_number_of_Internet_users
